# Syringomyelia associated to rheumatoid-atlantoaxial subluxation: about a new case

**DOI:** 10.11604/pamj.2017.28.158.13403

**Published:** 2017-10-19

**Authors:** Dhia Kaffel, Wafa Hamdi

**Affiliations:** 1 Rheumatology Department, Kassab Institute, Manouba, Tunisia

**Keywords:** Syringomyelia, atlantoaxial subluxation, rheumatoid arthritis

## Image in medicine

We report a case of 64-year-old Tunisian woman with a 12-year history of rheumatoid arthritis, who presented with a 3-month history of increasing inflammatory neck pain. Neurological examination noted a quadripyramidal syndrome without any neurological deficit. Radiographs of her neck showed ananterior atlantoaxial subluxation (A). MRI revealed a pannus around the atlanto axial joint. It also showed a co-existing syringomyelia (B). The cervical spine is frequently involved in patients with rheumatoid arthritis. However, rheumatoid atlantoaxial subluxation with syringomyelia is very rare. Many hypothesised mechanisms were suggested for the syrinx formation. The most commonly admitted, suggested that atlanto axial subluxation may reduce the rate of ascending cerebrospinal fluid, so it would travel through the spinal cord producing syringomyelia.

**Figure 1:**
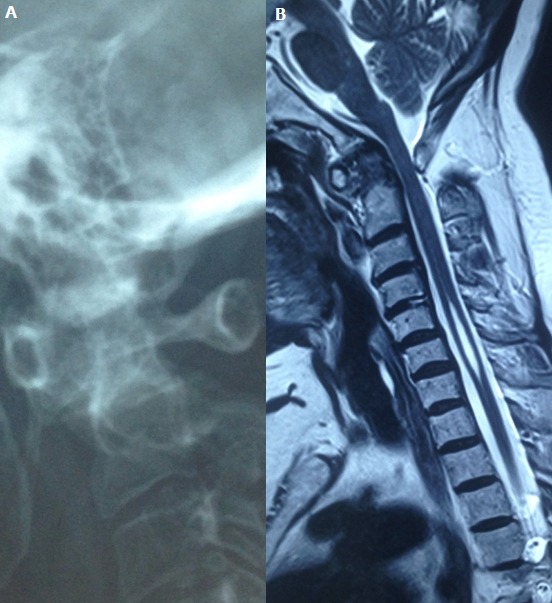
(A) radiograph of a cervical spine showing ananterior atlantoaxial subluxation; (B) MRI showing a pannus around the atlantoaxial joint with a co-existing syringomyelia

